# London

**Published:** 1855-12

**Authors:** 


### PROGRESS OF EPIDEMICS.

### LONDON.

The following is an abstract of the statistics of Epidemics occurring in London during the months of June, July, August, September, and October, 1855, as presented in the Registrar-General's Report: —

*Scarlet Fever.*—The mortality from this disease was in most weeks above the average of the corresponding weeks of the ten previous years, though generally much below the mortality of the corresponding week of 1854. The highest mortality was in the week ending October 20, being 76; the lowest that ending August 11, being 33. The southern districts suffered most severely, having been in the week ending July 21, 17, whilst all the other districts together only numbered 18.

*Measles.*—The deaths from this disease were every week greatly below the average of the same week in the ten previous years, as likewise below the deaths of the corresponding week in 1854. One week the deaths were 3 only, being 1 in each of the northern, central, and eastern districts, a mortality less than which is shown in but two weeks of the months corresponding to those summarized during ten previous years, those weeks being the ones ending July 4, 1846, and September 18, 1852, in both which the deaths were 2. During the 4 weeks of November, the numbers were 11, 15, 13, 20. The average of the same week of the previous ten years, as that in which the last of these numbers is shown, is 30·8, and of the mortality of the same week of 1854, 33. In the week ending July 14, the deaths were 1 in each of the west, north, central, east, and south districts.

*Small-pox.*—The mortality during the twenty-six weeks of the period was as follows: 24, 22, 27, 28, 24, 22, 18, 10, 13, 20, 9, 15, 12, 15, 14, 20, 11, 17, 20, 16, 16, 10, 14, 9, 14, 12. In the last week of November it was below the average of the same week of ten preceding years, and below the deaths of the same week in 1854; but it exceeded in most weeks the ten years average, considerably so up to July 7. The northern and southern districts, perhaps, suffered most, and the western least. During the six weeks ending November 24, however, only 1 death took place in the central district.

*Hooping Cough* was fatal in 794 cases, the greatest number of deaths (viz. 43,) occurring in the week ending June 9; and the least in the week ending September 22. In the week ending October 20, the deaths were 36; and of this number the southern districts, where indeed the mortality from this disease is generally greatest, furnished 16.

*Croup.*—The deaths from croup varied from 2 to 14; the total number having been 221.

*Influenza* presented a mortality never exceeding 2, and in twelve weeks no deaths were recorded. The total fatal cases were 19. Comparing this with the previous *three* months, we find that the deaths of the six months summarised only equal little more than half of the number (37) recorded in the three.

*Erysipelas.*—The deaths were generally lower than in the corresponding weeks of last year. In one week, that ending August 25, only 2 deaths took place.

*Ague* caused 11 deaths. In the three previous months there were 6 deaths. During the six weeks, August 18 to September 22, no deaths occurred.

*Remittent Fever.*—Starting the first week in June at 3, the deaths decreased to 0 in the week ending June 23; and again starting the week following at 3, they fluctuated between 3 and 1. This was the general variation during the previous three months.

*Diarrhœa.*—The deaths from diarrhœa during the first three weeks in June, were respectively 16, 16, 17. In the following week they were 25; and they increased, with a fall (15) one week only, till the week ending August 18, when the number was 154; the ten years average being 144·7, and the deaths of the corresponding week of 1854, 192. A nearly steady fall then took place till the last week in November, the number then having been 15. The total mortality of the previous three months was 192. Compared with the first three months of the six summarized, we find a great increase during the latter period; the mortality of *one week* being less than the total mortality of the former period by 38 only. Up to the week ending August 4, the deaths in the south districts were greatest, in most weeks, much the greatest; but in the week ending August 18, the deaths in the north districts were 45, and in the west only were the deaths less than in the south, the numbers being respectively 24, 25. In the week ending June 9, no deaths occurred in the east districts, and in the week ending November 24, none in the west.

*Dysentery.*—The deaths from dysentery vary between 0 and 6. The deaths decrease by 1 each week, for the four weeks, commencing with the week ending July 28, the numbers being 6, 5, 4, 3. In all 70 deaths took place. In the previous three months 30 deaths occurred.

*Typhus.*—The mortality from typhus was below that of the corresponding week in 1854, during the five weeks ending June 30; and also below during most of the following weeks. In the week ending November 24, however, the mortality was 54, that of the corresponding week of 1854 being 47, and the ten years average 53·4. The deaths from this disease may be roughly said to have been in inverse ratio to age, as the numbers show a descending progression as age increases.

*Puerperal Fever* was fatal in 81 cases; showing an improvement when compared with the 56 deaths in the previous three months. Still it was generally above the average, and highest in the week November 17, when 7 deaths occurred.

*Carbuncle.*—Deaths 23, the numbers being mostly 1 and 2. In the previous three months 10 cases occurred.

The greater number of deaths from each disease were in persons under 20 years of age.

---

### THE PROPAGATION OF CHOLERA.

